# Evaluation of Serum Homocysteine and High-Sensitivity Cardiac Troponins in Cats with Hepatic Lipidosis: An Observational Clinical Study

**DOI:** 10.3390/vetsci13050413

**Published:** 2026-04-23

**Authors:** Ahmet Cihat Tunç, Cemalettin Ayvazoğlu, Şemistan Kızıltepe, Sercan Hüseyin Bayendur, Abuzer Acar

**Affiliations:** 1 Department of Internal Medicine, Faculty of Veterinary Medicine, Afyon Kocatepe University, 03200 Afyonkarahisar, Turkey; cihattunc@aku.edu.tr (A.C.T.); sercanbayendurrr@gmail.com (S.H.B.); 2 Department of Laboratory and Veterinary Health, Nihat Delibalta Göle Vocational School, Ardahan University, 75002 Ardahan, Turkey; cemalettinayvazoglu@ardahan.edu.tr; 3 Department of Laboratory and Veterinary Health, Tuzluca Vocational School, Iğdır University, 76000 Iğdır, Turkey; semistan.kiziltepe@igdir.edu.tr

**Keywords:** feline hepatic lipidosis, serum homocysteine, hs-cTnI, hs-cTnT, hospitalization time, prognostic biomarker, subclinical myocardial stress

## Abstract

Feline hepatic lipidosis (FHL) is a life-threatening, common hepatobiliary disease characterized by massive triglyceride accumulation in the liver, often triggered by anorexia and negative energy balance in cats. This condition causes severe metabolic stress that may secondarily impact myocardial integrity. This observational clinical study evaluated serum homocysteine (Hcy) and high-sensitivity cardiac troponins (hs-cTnI, hs-cTnT) to assess secondary myocardial injury and their prognostic value in FHL. Fifty cats, comprising 30 with naturally occurring FHL and 20 healthy controls, were included. Serum Hcy, hs-cTnI, and hs-cTnT concentrations were measured using feline-specific ELISA kits, and routine biochemical parameters, alongside hospitalization times, were recorded. Results indicated that Hcy, total bilirubin, and liver enzymes were significantly elevated in the FHL group compared to controls (*p* < 0.001). However, hs-cTnI and hs-cTnT levels did not differ significantly between the groups. Notably, Hcy exhibited a strong positive correlation with hospitalization time (r = 0.89, *p* < 0.001). The absence of significant hs-cTn elevations suggests the feline myocardium remains largely resistant to acute necrosis during FHL. Nevertheless, relative hyperhomocysteinemia is a powerful prognostic biomarker for prolonged hospitalization in affected cats.

## Simple Summary

Feline fatty liver disease is a common and dangerous condition in cats that occurs when they stop eating, leading to rapid fat buildup in the liver. Because the liver is connected to many other systems in the body, this severe illness might also affect the heart. In this study, we aimed to determine whether cats with fatty liver disease exhibit heart muscle damage and how the severity of their condition impacts recovery time. We compared sick cats to healthy cats by measuring specific proteins and molecules in their blood that indicate heart stress and cellular damage. Our findings showed that the heart muscle of cats is surprisingly resistant to permanent damage from this liver disease. However, we discovered that a specific molecule, homocysteine, was much higher in sick cats. More importantly, cats with higher levels of this molecule had to stay at the animal hospital for much longer. Measuring this molecule could help veterinarians predict how long a sick cat will need intensive care, enabling better, earlier treatment planning.

## 1. Introduction

Feline hepatic lipidosis (FHL) is the most frequently encountered hepatobiliary disease in cats, characterized by a greater than 50% increase in liver mass and excessive triglyceride accumulation in over 80% of hepatocytes [1,2,3,4]. This life-threatening syndrome is typically triggered by a negative energy balance resulting from anorexia and starvation, which arise secondary to stress factors, environmental changes, or an underlying illness, predominantly in obese or overweight cats [5,6,7,8]. However, while increased body condition score and adiposity elevate the risk of FHL, it has been demonstrated that even cats with a normal body condition can develop mild hepatic lipidosis [8,9,10]. The clinical presentation predominantly features severe weight loss exceeding 25% of body weight, icterus, dehydration, vomiting, lethargy, and pronounced hepatomegaly. Without early interventions such as aggressive nutritional support, the mortality rate of the disease can exceed 90% [1,7,11,12]. A concurrent underlying primary condition, such as pancreatitis, gastrointestinal disorders, or cholangitis, is identified in 50% to 95% of FHL cases [5,10,13,14,15,16]. In biochemical evaluations, marked elevations in serum alkaline phosphatase (ALP) and alanine aminotransferase (ALT) activities are observed. In contrast, normal or only slightly elevated gamma-glutamyl transferase (GGT) activity is a crucial finding for the differential diagnosis of FHL [1,3,4,17]. At the cellular level, a hallmark characteristic distinguishing FHL from other hepatic diseases is the presence of minimal to no inflammation [7,18,19].

The complex pathophysiology of FHL is directly related to the unique protein and lipid metabolism of cats, which are obligate carnivores [1,20,21]. Relative insulin deficiency and insulin resistance, which develop secondarily during anorexia, stimulate lipolysis in peripheral adipose tissue, leading to the rapid mobilization of free fatty acids to the liver [6,7,22,23]. Under normal conditions, fatty acids that reach the liver should be converted into energy via mitochondrial beta-oxidation or secreted into the bloodstream as very-low-density lipoproteins (VLDLs) [7,17,24]. However, prolonged starvation in cats results in deficiencies of essential methyl-donor nutrients—such as L-carnitine, taurine, methionine, and B vitamins, which play a critical role in one-carbon metabolism—thereby disrupting fatty acid oxidation and VLDL secretion [7,21]. Unlike in humans, de novo lipogenesis in cats occurs primarily in visceral adipose tissue using acetate rather than in the liver; the subsequent transport of these newly synthesized lipids to the liver renders the triglyceride burden within hepatocytes even more intractable [2,7,25]. Recent metabolomic studies confirm at the cellular level that FHL is accompanied by increased hepatic beta-oxidation and disruptions in vitamin metabolism; furthermore, they indicate that innovative biomarkers reflecting this complex metabolic stress in the bloodstream could be highly valuable in elucidating the severity and pathogenesis of the disease [6,11,12,26].

In FHL, the unique one-carbon and protein metabolism inherent to feline obligate carnivores plays a critical role [4,20,27]. Because cats require significantly higher levels of sulfur-containing amino acids (e.g., methionine and cysteine) and cofactors (e.g., vitamin B6) than other mammals, plasma homocysteine reference intervals in healthy cats are notably higher and exhibit a broader distribution compared to other species [27,28,29]. Homocysteine, a key intermediate in methionine metabolism, is normally detoxified in the liver via folate- and cobalamin-dependent remethylation, alongside vitamin B6-dependent transsulfuration pathways [8,30,31]. However, the severe anorexia and hepatic dysfunction characteristic of FHL pathogenesis predispose patients to a deficiency of these sulfur-containing amino acids and essential cofactors vital for feline physiology [1,2,3,4]. The consequent malnutrition and secondary B-vitamin deficiencies disrupt these metabolic cycles, ultimately leading to homocysteine accumulation in the bloodstream [32,33,34]. Elevated homocysteine levels induce structural protein degradation, stimulate pro-inflammatory cytokines, and trigger severe cellular oxidative stress by increasing reactive oxygen species [31,35,36]. This metabolic toxicity causes cellular damage not only in the vascular endothelium but also systemically, potentially exerting deleterious effects on the cardiovascular system [28,35,37]. Despite these known mechanisms, the specific relationship between hepatic disease severity and homocysteine accumulation in cats remains incompletely elucidated in the veterinary literature.

Cardiac troponins are widely recognized as the gold standard for detecting myocardial injury, with high sensitivity and specificity. Upon cardiomyocyte damage or destruction, these globular proteins are released into the circulation, enabling the quantitative assessment of myocardial damage [38,39,40]. While conventional assays exhibit high limits of detection—often failing to measure baseline levels in healthy individuals and yielding primarily qualitative results—technological advancements have introduced high-sensitivity cardiac troponin (hs-cTn) assays that permit the precise, quantitative determination of minute concentrations [39,40,41,42]. With their significantly lowered detection limits compared to traditional methods, hs-cTnI and hs-cTnT measurements facilitate the identification of subtle, subclinical myocardial injuries that emerge both during the early stages of primary cardiac diseases and secondary to non-cardiac critical illnesses [39,40,41,42,43,44,45]. Consequently, the ability to capture even minor cellular perturbations establishes these high-sensitivity assays as robust diagnostic and prognostic tools for evaluating the cardiovascular impact of systemic diseases [39,40,43,44,45].

Therefore, the primary objective of the present study was to quantify serum homocysteine concentrations in cats diagnosed with FHL and to evaluate the potential association of this metabolite with high-sensitivity cardiac troponins I and T (hs-cTnI and hs-cTnT), the gold-standard biomarkers for cellular myocardial injury. By investigating the subclinical and secondary effects of FHL on the myocardium, this research aims to provide a novel perspective on the systemic pathophysiology of the disease.

## 2. Materials and Methods

### 2.1. Study Design and Ethical Approval

This prospective, cross-sectional, observational clinical study was conducted in full compliance with the ARRIVE guidelines. The study protocol was reviewed and approved by the Local Ethics Committee for Animal Experiments of Afyon Kocatepe University (Approval Date: 12 February 2025; Approval No: 49533702/286). All animals enrolled in the study were selected from naturally occurring clinical cases presented to and treated at the Veterinary Health Practice and Research Center of Afyon Kocatepe University between March 2025 and December 2025. Prior to study initiation, informed written consent was obtained from all owners, permitting the use of their pets’ clinical data and residual blood samples for research purposes. Blood samples were collected exclusively during routine venipuncture required for clinical diagnosis and therapeutic monitoring; no additional invasive procedures were performed solely for research purposes.

### 2.2. Study Population and Selection Criteria

A total of 50 cats were enrolled in this study, divided into a feline hepatic lipidosis (FHL) group (*n* = 30) and a healthy control group (*n* = 20). Cats assigned to the FHL group were selected from patients presenting with characteristic clinical signs, including severe anorexia, rapid weight loss, hepatomegaly, and icterus. Their biochemical profiles showed elevated alkaline phosphatase and total bilirubin levels, concurrent with normal gamma-glutamyl transferase activity, consistent with the FHL presentation. Abdominal ultrasonography, performed to support the presumptive diagnosis, revealed diffusely increased echogenicity of the hepatic parenchyma compared to the right renal cortex and spleen, rounding of the liver margins due to hepatomegaly, and reduced clarity of the intrahepatic portal vessel walls. To conclusively determine that these characteristic ultrasonographic findings stemmed from cellular lipid vacuolization, cytological examinations were conducted. Because the patients were poor clinical candidates for anesthesia and in consideration of ethical constraints, tissue biopsies were omitted; instead, ultrasound-guided fine-needle aspiration (FNA) was preferred [5,46]. The FNA procedure was performed using a 22-G hypodermic needle—selected to optimize cellular yield and preserve the integrity of lipid-laden hepatocytes—via the capillary technique from at least two distinct hepatic regions [47]. The resulting smears were stained with Diff-Quik, and the diagnosis of FHL was definitively confirmed by the presence of prominent vacuolar changes in the cytoplasm of more than 80% of hepatocytes, aligning with established literature criteria for severe lipidosis [18,19,48]. Following the procedure, vitamin K_1_ was administered as part of the standardized therapeutic protocol [1], and no clinical complications or persistent bleeding were observed during subsequent clinical monitoring.

The healthy control group consisted of cats with accessible previous medical records, no known systemic diseases, and body condition scores comparable to those of the FHL group prior to disease onset (4/5 scale). For all cats in this cohort, complete blood count, serum biochemistry, abdominal ultrasonography, and echocardiographic examination findings were evaluated and confirmed to be entirely within normal limits.

Stringent exclusion criteria were implemented to rule out potential comorbidities that could interfere with the clearance or synthesis of the evaluated cardiac biomarkers and homocysteine. Accordingly, cats suspected of having primary or secondary cardiac disease based on detailed echocardiographic examinations, those presenting with renal dysfunction indicated by elevated serum symmetric dimethylarginine (SDMA) concentrations, and those suspected of having triaditis due to increased feline-specific pancreatic lipase immunoreactivity (fPLI) were strictly excluded from the study. For all enrolled patients, a single venous blood sample was collected before initiation of any medical therapy. Standardized therapeutic protocols reported in the literature [1] were subsequently administered, and the duration of hospitalization until successful clinical recovery and discharge was recorded. To prevent survival bias in the analysis of hospitalization times, patients that died or were euthanized prior to completing the recovery process were entirely excluded from the dataset.

### 2.3. Blood Sampling and Laboratory Analysis

Blood samples were collected via standard venipuncture from the vena cephalica antebrachii or vena jugularis into plain tubes without anticoagulant. After collection, the samples were allowed to clot, and serum was separated by centrifugation at 4500× *g* rpm for 10 min. Routine biochemical variables, including ALT, AST, ALP, GGT, and total bilirubin, were measured immediately using an automated chemistry analyzer (Randox RX Daytona, Crumlin, United Kingdom; Serial No: 7241-0388).

To preserve biomarker stability, the remaining serum was aliquoted within 30 min of separation and immediately stored at –80 °C. To strictly prevent pre-analytical degradation, ELISA testing was performed in sequential batches throughout the study period, ensuring that the storage time for each sample did not exceed 3 months.

Serum homocysteine (Cat. No: SL0083FE), hs-cTnI (Cat. No: SL0084FE), and hs-cTnT (Cat. No: SL0085FE) concentrations were quantified using strictly feline-specific commercial ELISA kits (Sunlong Biotech, Hangzhou, China). To verify the analytical performance and reliability of the assays for feline serum, dilutional linearity and intra-assay precision were assessed using a randomly selected subset of 10 high-concentration samples. The observed linear recovery and low intra-assay coefficient of variation supported the excellent suitability and reproducibility of these assays for the study population. Absorbance values were read at 450 nm using a microplate reader (Thermo Scientific Multiskan GO, Vantaa, Finland; Serial No: 1510-04051C).

### 2.4. Statistical Analysis

Statistical analyses were conducted using the Python programming language (Pandas, SciPy v1.10, and Seaborn libraries). Descriptive statistics were computed for all continuous and categorical variables. The assumption of normality for continuous data was assessed using the Shapiro–Wilk test and visual inspection methods. For intergroup comparisons, appropriate statistical tests were selected based on data distribution and homogeneity of variance. Normally distributed variables (age and homocysteine) were compared using the independent samples *t*-test and are presented as the mean ± standard deviation (SD). Non-normally distributed variables or those lacking homogeneity of variance (BCS, hs-cTnI, hs-cTnT, total bilirubin, ALP, ALT, and AST) were analyzed using the non-parametric Mann–Whitney U test, with results expressed as the median and interquartile range (Q1–Q3). Intergroup comparisons of categorical data (breed distribution) were performed using the Chi-square test and are reported as frequencies (*n*). To maintain data integrity and robust statistical analysis, biomarker concentrations falling below the limit of detection (LOD) were substituted with LOD/√2, in accordance with established literature. Correlations among biomarker concentrations, clinical parameters, and hospitalization time were evaluated using Spearman’s rank correlation coefficient (rho), accounting for the data distribution. For all analyses, a *p*-value < 0.05 was considered statistically significant.

### 2.5. The Use of Artificial Intelligence for Linguistic Refinement

During the preparation of this manuscript, the software tool Grammarly Premium (Institutional Version) was utilized strictly for grammar correction, linguistic refinement, and improving the overall readability of the text. No automated tools were used in data analysis, interpretation of the results, or the drawing of scientific conclusions. The authors thoroughly reviewed, edited, and approved all texts and take full and absolute responsibility for the final content of the publication.

## 3. Results

### 3.1. Demographic Characteristics of the Study

The demographic characteristics of the 50 enrolled cats are summarized in Table 1. No statistically significant differences were observed between the FHL and healthy control groups regarding age, body condition score, and breed distribution.

### 3.2. Comparison of Biomarkers and Biochemical Parameters

**Figure 1:**
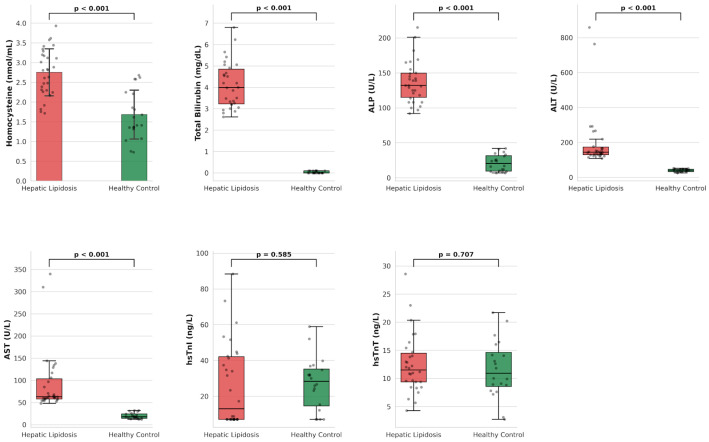
Bar and Box-and-Whisker plots illustrating the distributions of measured parameters across study groups. The boxes represent the interquartile range (Q1–Q3). The horizontal line within the box indicates the median value, and the whiskers extend to the minimum and maximum values (excluding outliers). Homocysteine and biochemical parameter levels were significantly elevated in the HL group compared with controls (*p* < 0.001). No statistically significant difference was observed in hs-cTnI and hs-cTnT levels.

Serum homocysteine concentrations in the FHL group were significantly higher compared to the healthy control group (*p* < 0.001). Similarly, liver injury markers, including Total Bilirubin, ALP, ALT, and AST, were significantly elevated in the patient group (*p* < 0.001 for all). In contrast, no statistically significant differences were observed between the groups regarding the cardiac biomarkers hs-cTnI and hs-cTnT (*p* > 0.05). The group distributions for all evaluated biomarkers and biochemical parameters are presented in Table 2 and Figure 1.

### 3.3. Correlation Analysis

**Figure 2:**
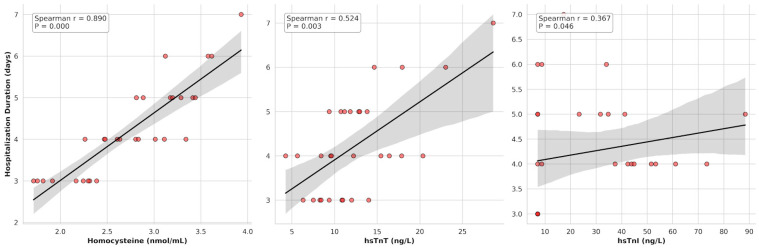
Scatter plots illustrating the significant positive correlations between hospitalization time and serum concentrations of homocysteine (r = 0.89, *p* < 0.001), hs-cTnT (r = 0.52, *p* = 0.003), and hs-cTnI (r = 0.37, *p* = 0.046) in the feline hepatic lipidosis group. Dots represent individual data points (cats), solid lines represent the linear regression of the data, and the shaded areas denote the 95% confidence intervals.

The associations between clinical and biochemical parameters and hospitalization time within the FHL group were assessed using Spearman’s rank correlation analysis. A remarkably strong positive correlation was identified between serum homocysteine concentrations and hospitalization time (r = 0.89, *p* < 0.001). Regarding cardiac biomarkers, moderate and weak-to-moderate significant positive correlations were observed between hospitalization time and hs-cTnT (r = 0.52, *p* = 0.003) and hs-cTnI (r = 0.37, *p* = 0.046), respectively (Figure 2). In contrast, no statistically significant associations were found between hospitalization time and the levels of total bilirubin, ALT, or ALP (*p* > 0.05).

**Figure 3:**
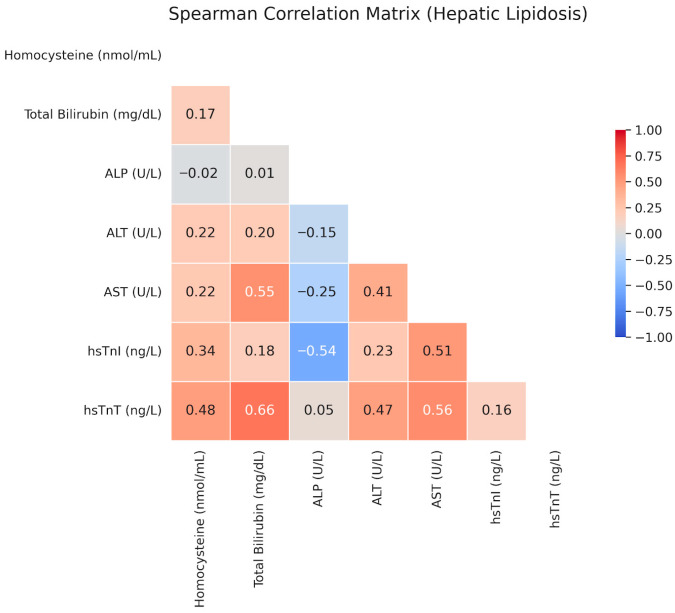
Spearman’s rank correlation matrix of clinicopathological variables, cardiac biomarkers, and hospitalization time in cats with feline hepatic lipidosis. The color gradient indicates the strength and direction of the correlation coefficients.

Upon examining the interrelationships among the evaluated parameters within the FHL group, hs-cTnT levels demonstrated significant positive correlations with total bilirubin (r = 0.66, *p* < 0.05) and AST (r = 0.56, *p* < 0.05). Additionally, a significant negative correlation was identified between hs-cTnI levels and ALP (r = −0.54, *p* < 0.05). Scatter plots illustrating these correlations are presented in Figure 3.

## 4. Discussion

To the best of our knowledge, this is the first study to simultaneously evaluate the prognostic value of serum homocysteine and the impact of the FHL-induced metabolic milieu on myocardial integrity utilizing high-sensitivity biomarkers. Our findings highlight two fundamental clinical concepts. First, relative hyperhomocysteinemia is not merely a prominent feature of FHL; its remarkably strong positive correlation with hospitalization time strongly suggests its utility as a potent prognostic biomarker. Second, despite the profound stress, severe cholestasis, and metabolic burden characteristic of the disease, the absence of significant elevations in high-sensitivity cardiac troponin levels compared with healthy controls indicates that the feline myocardium remains largely resistant to acute necrotic injury. Nevertheless, our data also indicate specific interactions between hepatic metabolic burden and subclinical myocardial cellular leakage.

In the present study, while no statistically significant elevations in hs-cTn levels were observed among cats with FHL, Hcy concentrations were significantly increased and correlated positively with hs-cTnT. Although highly specific to myocardial tissue, cardiac troponins can be elevated not only in primary cardiac diseases but also across a diverse spectrum of non-cardiac systemic conditions, including sepsis, systemic inflammatory response syndrome (SIRS), and renal failure [49,50,51]. Troponin release in these non-cardiac contexts is not invariably associated with irreversible cell necrosis; rather, it can also be driven by mechanisms of reversible ischemia and cellular stress, such as increased cell membrane permeability, oxidative stress, or the presence of inflammatory cytokines [50,51,52]. Furthermore, hs-cTn assays are exceptionally sensitive biomarkers capable of detecting even microscopic or low-grade myocardial injury, representing a crucial milestone in clinical risk stratification [49,50,53]. Given this high analytical sensitivity, the lack of statistically significant troponin elevations in the FHL cohort strongly suggests the absence of acute and severe myocardial necrosis. Nevertheless, the concurrent significant elevation in Hcy levels and its moderate positive correlation with hs-cTnT remain highly noteworthy.

It has been comprehensively established that Hcy exerts direct toxic effects on the cardiovascular system at the cellular level by triggering pathways such as oxidative stress, endothelial dysfunction, endoplasmic reticulum stress, and apoptosis/pyroptosis [37,52,53]. Experimental models have shown that hyperhomocysteinemia can lead to pathological ventricular hypertrophy, increased myocardial fibrosis, and diastolic dysfunction, independent of other known hemodynamic stimuli, while also directly suppressing myocardial contractile function via endothelium-mediated mechanisms [54,55]. This pathophysiological link between Hcy and troponins is further supported by clinical studies showing that elevated Hcy concentrations are directly and positively correlated with subclinical myocardial injury and increased troponin levels [53]. Consequently, the correlation between Hcy and hs-cTnT identified in our study indicates that the systemic metabolic stress and elevated Hcy levels induced by hepatic lipidosis in cats can provoke myocardial stress, even in the absence of severe necrosis. These findings underscore the clinical importance of combining Hcy—reflecting cellular dysfunction and vascular stress—and hs-cTn assays—detecting microscopic myocardial injury—to elucidate secondary cardiac effects in systemic diseases such as hepatic lipidosis.

It is well established that in severe hepatic diseases and cholestatic conditions, bile acids and bilirubin exert direct toxic effects on cardiomyocytes, disrupting cell membrane fluidity and permeability [56,57,58]. Although hs-cTn levels were not significantly elevated in the FHL group in our study, intragroup analyses revealed strong to moderate positive correlations between hs-cTnT and both total bilirubin and AST. This finding suggests that even in the absence of clinically significant ischemia, the escalating cholestatic and toxic burden can induce subclinical stress and cytosolic leakage across the cardiomyocyte membrane, mirroring a dose–response relationship [59]. As is widely recognized, hs-cTnT and hs-cTnI exhibit distinct release dynamics and reflect different underlying pathologies. Because it is more tightly bound to tropomyosin within cardiomyocytes, hs-cTnT serves as a more robust indicator of chronic processes involving compromised cellular integrity, generalized cardiovascular stress, and non-ischemic systemic morbidities, rather than reflecting acute ischemic necrosis [60]. The specific correlation of elevated total bilirubin and AST with hs-cTnT indicates that the toxic milieu induced by FHL generates generalized systemic membrane stress in cardiomyocytes without culminating in acute necrosis. Furthermore, in contrast to the structural degradation anticipated in classical ischemic myocardial injury, our study identified a moderate negative correlation between ALP and hs-cTnI. It has been reported that hs-cTnI is vastly more sensitive to acute ischemic myocardial injury than hs-cTnT, undergoing rapid proteolytic degradation and subsequent release into the circulation upon necrosis [60]. In the setting of severe cholestasis characterized by marked ALP elevation, the absence of an increase—and indeed, the presence of a negative correlation—in hs-cTnI, the primary marker of acute ischemic injury, suggests that the feline heart exhibits remarkable structural adaptation to this cholestatic toxicity, potentially preventing the progression to acute ischemic necrosis.

In the present study, Hcy levels were significantly elevated in cats with FHL compared with the healthy control group, and a remarkably strong positive correlation was observed between increasing Hcy concentrations and hospitalization time. Clinical investigations across various disease models have demonstrated that hyperhomocysteinemia serves as an independent risk factor predisposing patients to prolonged hospitalization, extended intensive care unit stays, and increased overall morbidity [61,62,63]. In light of these data, it is plausible that the elevated homocysteine levels observed in our cohort constitute a major factor directly prolonging hospitalization and complicating the clinical recovery of patients with FHL. Physiologically, both folate and cobalamin act as essential cofactors involved in the remethylation of homocysteine to methionine; consequently, deficiencies in either of these vitamins disrupt this metabolic pathway, ultimately culminating in hyperhomocysteinemia [21,64]. Felines possess a notably limited capacity for cobalamin storage, and although severe folate deficiency is less common, serum concentrations can also drop significantly in cases of severe malabsorption. Furthermore, it has been widely reported that concurrent enteropathies frequently complicate FHL, predisposing these patients to profound cobalamin and potential folate deficiencies due to malabsorption [21,65,66,67]. A limitation of the present study is the absence of direct cobalamin and folate measurements. While we utilized normal fPLI levels to exclude severe comorbidities like triaditis, in retrospect, we acknowledge that subclinical or occult concurrent enteropathies could have remained clinically undetected. Therefore, when evaluating these established pathophysiological mechanisms collectively, the homocysteine elevation observed in the FHL group is probably driven by a concurrent, underlying cobalamin and/or folate deficiency, which is frequently encountered in these patients [21,64,66,67]. Accordingly, therapeutic cobalamin and folate supplementation in patients with FHL could help regulate homocysteine metabolism, thereby facilitating recovery and potentially reducing prolonged hospitalization. Nevertheless, because direct concentrations were not quantified in our cohort, this potential therapeutic efficacy should be interpreted with caution. This hypothesis warrants validation through comprehensive future studies that concurrently evaluate homocysteine, cobalamin, and folate levels, while also carefully accounting for the potential impact of other concurrent diseases on hospitalization duration.

Interestingly, the absolute homocysteine concentrations in our cohort were lower than those reported in previous studies of healthy cats [27,29]. A primary factor contributing to this discrepancy is the variation in analytical methodologies. While our study utilized a feline-specific ELISA kit, previous investigations employed high-performance liquid chromatography-tandem mass spectrometry (HPLC-MS/MS) [27] or human-specific ELISA assays [29]. Furthermore, environmental and demographic dynamics can significantly influence baseline levels. It has been reported that feeding cats high-protein diets markedly elevates homocysteine concentrations [27,33], and breed-specific characteristics may result in distinct reference intervals [29]. Despite these methodological and population-based discrepancies in absolute values, identical standardized procedures were applied to both cohorts in our study. Therefore, the relative increase observed between the FHL and healthy control groups remains internally consistent and retains its profound clinical significance.

## 5. Conclusions

This study demonstrates that despite the severe toxic and metabolic burden induced by feline hepatic lipidosis (FHL), the feline myocardium exhibits remarkable structural resistance to acute ischemic necrosis, as evidenced by high-sensitivity cardiac troponins (hs-cTnI and hs-cTnT). However, the correlations between elevated homocysteine, bilirubin, and AST levels and hs-cTnT indicate that systemic toxicity, although not causing acute tissue destruction, leads to subclinical membrane stress and cellular leakage in cardiomyocytes. The most clinically striking outcome of the study is the very strong positive correlation between significantly elevated serum homocysteine concentrations and hospitalization time in patients with FHL. This finding highlights that homocysteine is a highly potent and innovative prognostic biomarker that can be used to predict disease course, extent of metabolic damage, and hospital length of stay at an early stage.

## Figures and Tables

**Table 1:** Comparison of demographic characteristics between the feline hepatic lipidosis (FHL) and healthy control groups.

Parameters	FHL Group (*n* = 30)	Healthy Control Group (*n* = 20)	*p*-Value
Age (Years)	4.93 ± 1.60	4.50 ± 1.10	0.296
Gender (Male/Female)	13/17	10/10	0.385
BCS	4.0 (4.0–4.0)	4.0 (3.8–4.0)	0.416
Breed Distribution (*n*)			0.342
Domestic Shorthair	17	13	
British Shorthair	10	7	
Siamese	3	0	

**Table 2:** Comparison of serum biochemical parameters, homocysteine, and high-sensitivity cardiac troponin (hs-cTn) levels between cats with hepatic lipidosis (FHL) and the healthy control group.

Parameters	FHL Group (*n* = 30)	Healthy Control Group (*n* = 20)	*p*-Value
hs-TnI (ng/L)	13.00 (7.07–42.14)	28.33 (14.59–35.30)	0.585
hs-TnT (ng/L)	11.50 (9.38–14.49)	10.91 (8.56–14.64)	0.707
Homocysteine (nmol/mL)	2.75 ± 0.60	1.68 ± 0.62	<0.001
Total Bilirubin (mg/dL)	3.99 (3.23–4.85)	0.00 (0.00–0.10)	<0.001
ALP (U/L)	132.0 (115.0–149.75)	20.50 (9.75–31.50)	<0.001
ALT (U/L)	143.50 (129.75–173.75)	40.50 (32.50–46.25)	<0.001
AST (U/L)	63.0 (58.0–103.75)	18.00 (14.75–24.50)	<0.001

hs-cTnI: High-sensitivity cardiac Troponin I, hs-cTnT: High-sensitivity cardiac Troponin T, ALP: Alkaline Phosphatase, ALT: Alanine Aminotransferase, AST: Aspartate Aminotransferase.

## Data Availability

The data presented in this study are available on request from the corresponding author due to ethical restrictions and the need to protect animal owner confidentiality.
